# A Novel Strategy to Mitigate the Hyperinflammatory Response to COVID-19 by Targeting Leukotrienes

**DOI:** 10.3389/fphar.2020.01214

**Published:** 2020-08-06

**Authors:** Colin D. Funk, Ali Ardakani

**Affiliations:** ^1^Department of Biomedical and Molecular Sciences, Queen’s University, Kingston, ON, Canada; ^2^Scientific Research Division, Novateur Ventures Inc., Vancouver, BC, Canada

**Keywords:** COVID-19, SARS-CoV-2, leukotrienes, cytokine storm, coronavirus, inflammatory response, vascular leak, clinical trial

## Abstract

SARS-CoV-2 causing coronavirus disease 2019 (COVID-19) has wreaked havoc during the global pandemic of 2020 infecting millions and leaving over a half million dead. As a new virus, not previously in the human population, but with similarities to other coronaviruses causing severe acute respiratory distress syndrome (SARS/ARDS), and no known treatments, the race to re-purpose existing drugs and to enlist novel therapeutics is underway. In the half-year since the first cases, we have acquired substantial knowledge of this virus and the clinical course of COVID-19 progression. Results from early clinical trials have revealed two treatments (remdesivir, dexamethasone) that mitigate disease progression but clearly, there is much room for improvement. Initial case reports indicated many succumb to COVID-19 of hypoxic respiratory failure due to ARDS. However, ensuing studies revealed an atypical, immune cell-sequestered, vasculature-inflamed state leading to multiorgan thrombotic complications and end organ failure likely due to hyperinflammatory host responses. This Perspective focuses on a potential mechanism for a key COVID-19 disease progression turning point related to vascular and airway inflammation. The leukotriene lipid mediators have been overlooked with discussion centering on cytokine storms unleashing the deadly form of COVID-19. Leukotrienes possess some of the most potent known activities on immune cell trafficking and vascular leakage. We offer a simple treatment paradigm using two generic drugs targeting the hyperinflammatory response that characterizes the turning point from mild to severe/critical COVID-19 by targeting leukotriene biosynthesis with zileuton (Zyflo^®^ controlled release formulation) and antagonism of the cysteinyl leukotriene 1 receptor with montelukast (Singulair^®^).

## Introduction

SARS-CoV-2 (Severe acute respiratory syndrome coronavirus 2) is the viral instigator of coronavirus disease 2019 (COVID-19) (Oberfeld et al., 2020; Wu F. et al., 2020; Zhou P. et al., 2020; Zhu et al., 2020). Striking in China in late 2019, the virus has spread to virtually every inhabited space on the globe creating a wave of infection killing over half a million people in the first 6 months of the pandemic^1^. This pneumonia-causing disorder spreads primarily from respiratory droplets of infected individuals in enclosed spaces to mucosal epithelial cells in the upper airway and oral cavity where it gains entry *via* its homotrimeric spike protein to host-cell expressed angiotensin-converting enzyme-2 (ACE2) receptor binding sites in a protease-dependent manner (Liu et al., 2020; Oberfeld et al., 2020). Viral RNA is released into the cytoplasm and hijacks the cell coordinating a replication-transcription complex, whereby viral RNAs are translated into a distinct set of proteins. Virion assembly is completed and the viral particles can perpetuate the cycle by infecting new cells in the lower airways (Type II pneumocytes), enterocytes in the gastrointestinal tract, while some eventually enter the bloodstream *via* damaged host tissues (e.g., alveoli) and will bind to ACE2 in vascular endothelial cells to initiate a cascade of deleterious events throughout the body (Lamers et al., 2020; Oberfeld et al., 2020; Varga et al., 2020).

## COVID-19 as a Hypoxia/ARDS Clinical Disorder With a Unique Vascular Hyperinflammatory/Procoagulant State

**Figure 1:**
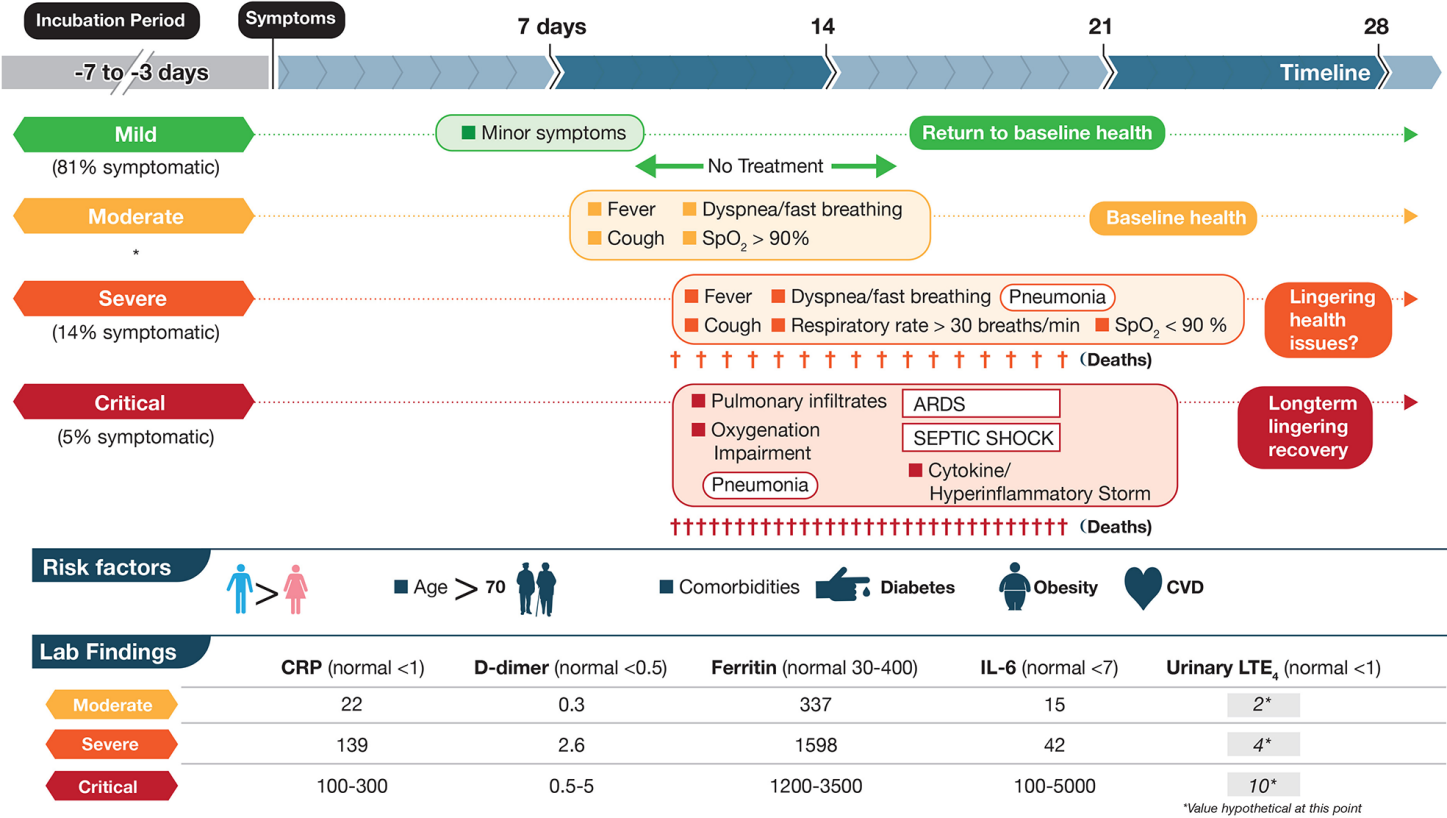
Schematic depictions of mild, moderate, severe, and critical cases of coronavirus disease 2019 (COVID-19) including some symptoms, laboratory findings, and clinical timeline. The disease course is highly variable with dotted lines representing this unpredictability. Criteria are based on World Health Organization categories. In a large cohort of COVID-19 patients that experience symptoms (mentioned in Berlin et al., 2020), percentages are shown in three categories mild, severe and critical. Moderate were not included (shown by *). Up to half of critical cases may die from COVID-19 complications with fewer deaths in the severe category. Lab data are based on (England et al., 2020; table 6) and are meant to show approximations for each marker. Units for CRP, D-dimer, Ferritin, IL-6 are mg/L, μg/ml, μg/L, and pg/ml, respectively. The numbers in the LTE_4_ column, at this point, are hypothetical and based on baseline data of urinary LTE_4_ levels of ≈1 ng/mg creatinine in normal controls and 4 ng/mg creatinine in severe ARDS patients (Bernard et al., 1991). These values would need to be validated in SARS-CoV-2 infected individuals. LTE_4_ is the major urinary metabolite of the cysteinyl leukotrienes LTC_4_ and LTD_4_.

**Figure 2:**
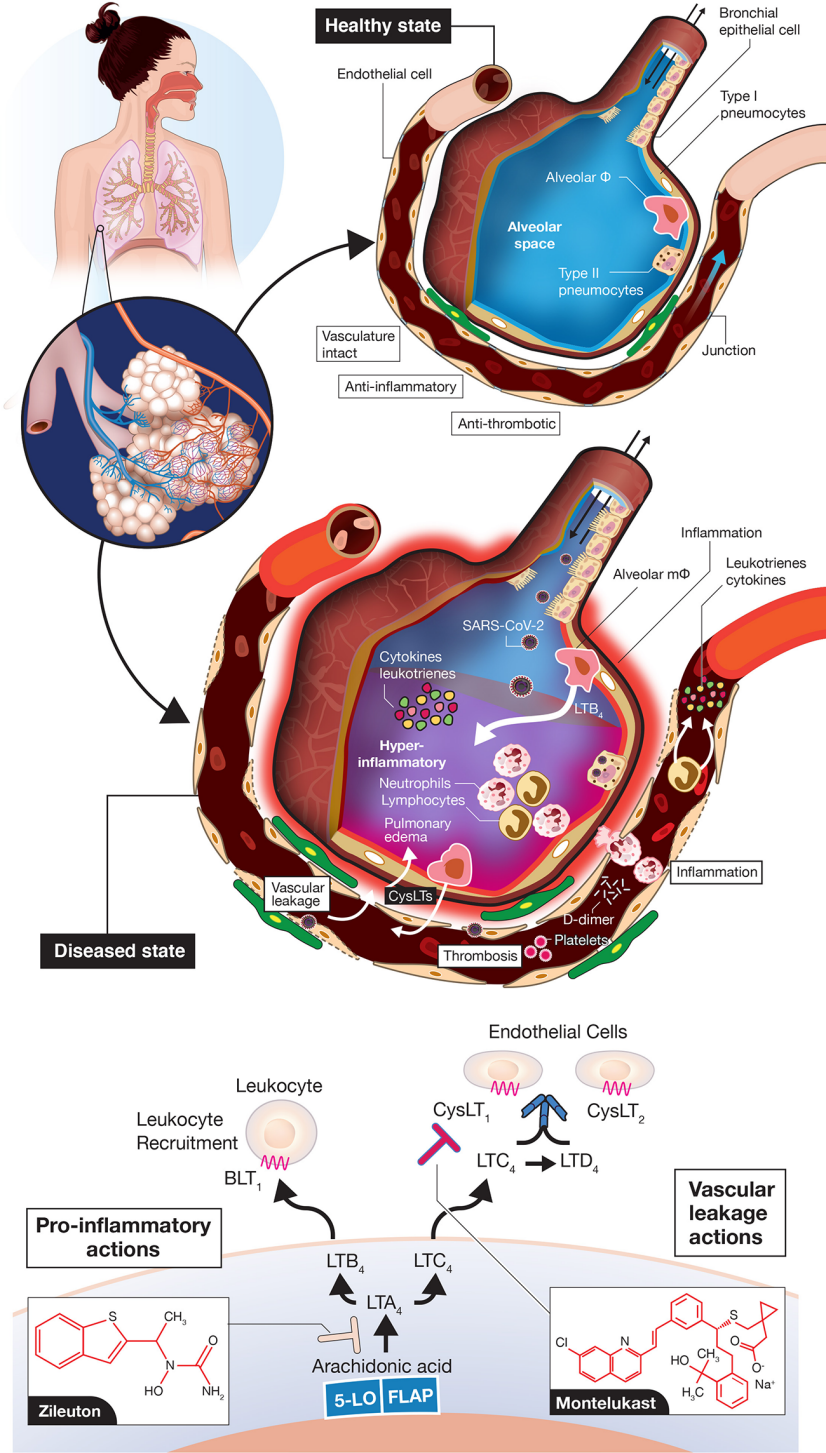
Vascular leakage, inflammation-provoking, and thrombotic events in coronavirus disease 2019 (COVID-19). A normal alveolus in the healthy state with associated capillary vessel (top) and SARS-CoV-2 infected alveolus with surrounding vasculature (middle) are shown. Potential roles for LTB_4_ to promote inflammatory cell influx into the airways and for CysLTs to initiate vascular leakage are depicted. LTB_4_ and CysLTs can be synthesized *de novo* by alveolar macrophages, infiltrating leukocytes, sentinel mucosal mast cells (not shown here) and *via* interactions of inflammatory cells with endothelial cells. The figures are “cartoon” representations, so cells/viruses/blood vessels are not to scale. Concepts of the model indicating that COVID-19 is an endothelial disorder are based on (Ackermann et al., 2020; Becker, 2020; Sardu et al., 2020; Teuwen et al., 2020; Varga et al., 2020) but with an emphasis here on leukotriene inflammatory mediators. Depiction of leukotriene biosynthesis along with the two drugs zileuton and montelukast proposed to mitigate disease progression of COVID-19 (bottom). Zileuton, acting intracellularly, inhibits 5-lipoxygenase (5-LO) to decrease leukotriene ligands able to bind downstream receptors BLT_1_ (mediating neutrophil/T lymphocyte trafficking) and CysLT receptors (promoting vascular leakage). Montelukast, acting extracellularly, antagonizes selectively CysLT_1_ to dampen inflammation and reduce vascular leakage. FLAP, 5-lipoxygenase-activating protein.

COVID-19 can be divided roughly into mild, moderate, severe, and critical cases (Berlin et al., 2020; Chen at al., 2020; **Figure 1**). The vast majority of infected individuals remain largely asymptomatic or only develop mild symptoms. These four general classifications may be part of a continuum in the same individual or discrete, distinct clinical entities in other cases that do not progress from one category to the next. Once infected with SARS-CoV-2, the median incubation period is 5 days to symptoms, although this can be significantly shorter or longer, presumably due to initial viral load of exposure (Berlin et al., 2020). Initial clinical symptoms include fever, dry and persistent cough, and fatigue with the potential for a wide range of other symptoms (e.g., loss of taste/smell, loss of appetite, dyspnea, headaches, sore throat, myalgia, intestinal discomfort/diarrhea, conjunctivitis) with extensive variations between adults and children (Guan et al., 2020; Oberfeld et al., 2020). Severe symptoms leading to hospitalization that progress rapidly to hypoxia and acute respiratory distress syndrome (ARDS) requiring supplemental oxygen and ventilator support are most prevalent in the elderly with underlying co-morbidities such as diabetes, obesity, and cardiovascular disease (Vetter et al., 2020; Du et al., 2020) (see **Figure 1**). Circulating cytokines, such as IL-6, IL-8, TNFα are significantly elevated in severe COVID-19 patients with documented SARS-CoV-2 infection in pneumocytes and endothelial cells, which leads to severe alveolar damage (**Figure 2**). In severe cases, lymphocytopenia is evident, with depletion of CD4^+^ and CD8^+^ lymphocyte subsets (Li et al., 2020) in blood. There is associated endothelial cell damage of pulmonary vessels with widespread thrombosis (elevated marker: D-dimer), complement activation and microangiopathy (Ackermann et al., 2020; Becker, 2020; Magro et al., 2020). The patients with severe COVID-19 develop an overwhelming state of inflammation (elevated marker: C-reactive protein) with multiorgan dysfunction that has been labeled COVID-19 cytokine storm syndrome (CSS) (England et al., 2020). Comparisons to other disorders including secondary hemophagocytic lymphohistiocytosis (sHLH), macrophage-activation syndrome (MAS), Castleman disease, and the cytokine-release syndrome (CRS) associated with chimeric antigen receptor T cell therapy (CAR-T)) have been detailed (Becker, 2020; England et al., 2020) but none match precisely what has been seen in COVID-19 CSS. An unusual presentation in children, similar to Kawasaki Disease, termed MIS-C (multisystem inflammatory disease in children) is showing enhanced prevalence (Viner and Whittaker, 2020). Increasingly clear is the unique multifocal nature of COVID-19 pathogenesis. SARS-CoV-2 may instigate destruction to blood vessel endothelial cells leading to coagulopathy and strokes, with ensuing damage to kidneys, perhaps pancreatic islet cells, along with neurological problems (Ackermann et al., 2020; Becker, 2020; Mallapaty, 2020; Sardu et al., 2020; Teuwen et al., 2020). Factors affecting disease severity and protective immunity include genetics, age, co-morbidities, sex, ethnicity, demographics, and likely many more that have not yet been defined (Casanova et al., 2020). Overall, SARS-CoV-2 appears to promulgate a novel clinical presentation never before seen in the human population.

## Mechanistic Pathways Explaining COVID-19 Progression That Involve Leukotrienes

While a majority of attention has focused on the COVID-19 CSS provoking an over exuberant host immune response to SARS-CoV-2 infection in severe/critical cases, some have used a more broad description of a hyperimmune or hyperinflammatory storm (Alunno et al., 2020; Becker, 2020; England et al., 2020; Jamilloux et al., 2020; Jose and Manuel, 2020; Mehta et al., 2020; Panigrahy et al., 2020). In this classification, other inflammation-provoking molecular entities, in addition to cytokines, would be included. Severe disease pathogenesis is likely the result of alveolar pneumocyte injury leading to SARS-CoV-2 dissemination to vascular endothelial cells facilitated by widespread ACE2 expression in vascular beds (Ackermann et al., 2020; Becker, 2020; Teuwen et al., 2020; Varga et al., 2020) (**Figure 2**). Breaches in vascular integrity have been reported with profound vascular leakage/permeability changes (Ackermann et al., 2020; Becker, 2020; Teuwen et al., 2020). These changes could also influence influx of inflammatory cells throughout the airways and vessel walls. A totally neglected set of molecules not discussed to date include the class of lipid mediators known as leukotrienes (Samuelsson et al., 1987; Funk, 2001; Capra et al., 2007; Peters-Golden and Henderson, 2007) (**Figure 2**). Leukotrienes are prime candidates to provoke the hyperimmune/inflammatory response in progressing COVID-19, with elevated leukotriene levels detected previously in tracheal aspirates of patients with ARDS (Sala et al., 1991). Leukotriene B_4_ (LTB_4_) is one of the most potent known chemoattractants for neutrophils (Ford-Hutchinson et al., 1980; Bisgaard et al., 1986) and lymphocyte subsets (Tager et al., 2003; Taube et al., 2006), *via* signaling through the B leukotriene subtype 1 (BLT_1_) G protein-coupled receptor (GPCR) (Sasaki and Yokomizo, 2019). LTB_4_ is likely one of the key mediators carrying out the huge influx of these cells to airways, which leads to the profound blood lymphocytopenia observed in severe COVID-19 and neutrophilia in airways (Zhang et al., 2020; Li et al., 2020). Moreover, the cysteinyl leukotrienes (cysLTs) LTC_4_ and LTD_4_ are among the most profound vascular leakage promoting agents in man and animal models, signaling *via* two GPCR subtypes CysLT_1_ and CysLT_2_ (Samuelsson et al., 1987; Funk, 2001; Maekawa et al., 2002; Lee et al., 2004; Moos et al., 2008; Capra et al., 2015). CysLTs also provoke a number of immune cell actions e.g., macrophage activation, inflammatory cell cytokine secretion and activation of the transcription factor NF-κB, which controls numerous genes involved in inflammation, all of which would heighten the hyperimmune/inflammatory response (Kanaoka and Boyce, 2004; Maeba et al., 2005; Tahan et al., 2008) in COVID-19.

If leukotrienes (LTs) have such potent inflammation-promoting actions, why have they not been considered so far? First, they are not easily measured compared to widely available clinical diagnostic assays of cytokines and other generalized biomarkers of inflammation (e.g., C-reactive protein, CRP) and coagulation (e.g., D-dimer), respectively. There are no routine diagnostic tests in hospitals and other clinical settings for measuring LTs. Their measurement most often entails a labor-intensive mass spectrometry assay with prior solid-phase extraction from blood or urine (Murphy et al., 2005), with most commercially available ELISA kits not recommended for human diagnostics. Second, lipid inflammatory mediators, in general, receive less attention than their cytokine counterparts due to their labile nature and rapid metabolism. Third, there are very few clinically approved drugs in the leukotriene modifier class (Funk, 2005; Capra et al., 2006; Werz and Steinhilber, 2006). The field to developing successful therapeutics has been fraught with a minefield of abandoned pre-clinical candidates. After 30^+^ years of targeted research in the field by many leading pharmaceutical companies, there are only a few approved drugs in the pathway^2^; one is the 5-lipoxygenase enzyme inhibitor, which blocks the synthesis of all downstream LTs, known as zileuton (Zyflo^®^) (Werz and Steinhilber, 2006; Bouchette and Preuss, 2020) and the other is an antagonist of the CysLT_1_ receptor, montelukast (Singulair^®^; with two other approved drugs in this class) (Wermuth et al., 2020). No drugs have reached the clinical market for other targets in the leukotriene pathway (**Figure 2**, bottom) including the 5-lipoxygenase-activating protein (FLAP), leukotriene A_4_ hydrolase, two subtypes of B leukotriene receptors (BLT_1_, BLT_2_) and for CysLT_2_, although many pre-clinical candidates have been advanced over the years (Funk, 2005; Werz and Steinhilber, 2006).

## Dual Drug Treatment Paradigm for COVID-19 Targeting Leukotrienes

There are currently no approved effective therapies or preventative vaccines to protect the immune naïve global population from COVID-19. Over 2500 clinical trials are registered worldwide in attempts to treat the clinical sequelae of SARS-CoV-2 infection^3^, ^4^. Numerous drugs, both approved or in preclinical development, designed to treat other disorders have been repurposed to treat COVID-19 in the first half of 2020. Several drug candidates have already been ruled out as effective agents, based on early trial results (e.g., hydroxychloroquine), with only two (the antiviral remdesivir and the synthetic glucocorticoid dexamethasone) showing partial efficacy in randomized clinical trials (Goldman et al., 2020; Recovery Collaborative Group, 2020; Wang et al., 2020). Current WHO guidelines (as of June 30, 2020) do not recommend treatment with any drugs outside of clinical trials, although some regional authorities are now incorporating dexamethasone into treatment guidelines to combat the hyperinflammatory stages of severe COVID (Mahase, 2020).

At the time of writing this perspective, we have found only three papers out of >20,000 listed on PubMed mentioning COVID, that hypothesize the use of a clinically approved drug targeting leukotrienes, known as leukotriene modifiers (Funk, 2005), namely the leukotriene receptor antagonist (LTRA) montelukast for COVID-19 treatment (Almerie and Kerrigan, 2020; Bozek and Winterstein, 2020; Fidan and Aydoğdu, 2020). In addition, the first and only registered trial mentioning leukotrienes was just recently registered (May 15, 2020) - The COvid-19 Symptom MOntelukast Trial (COSMO)^5^. While these are welcome forays into the area, we believe for optimal chances to relieve the hyperimmune/inflammatory storm in COVID-19 it will be necessary to not only block CysLT_1_ signaling with montelukast but also the other LT receptors, CysLT_2_ and BLT_1_. Since there are no approved blockers for these latter two receptors, it would be imperative to reduce production of all LTs from inflammatory cells before the deadly orchestration of hyperinflammation and cascade of procoagulant actions can take place. This could be achieved potentially with both a 5-lipoxygenase inhibitor (zileuton) and an LTRA (montelukast). Montelukast has been in widespread use for over 20 years to treat the airway inflammatory symptoms of mild-moderate asthma and allergic rhinitis and has an excellent safety profile (Jones et al., 1995; Wermuth et al., 2020). However, zileuton, also used for the same indication and on the market for >20 years (Rubin et al., 1991; Bouchette and Preuss, 2020), has been used much less frequently mainly due to weaker potency (large 600 mg tablets) and poor pharmacokinetics (increased dosing) with the potential for hepatotoxicity (Funk, 2005; Werz and Steinhilber, 2006; Bouchette and Preuss, 2020). The newer controlled release (CR) formulation (two tablets, bid) obviates partially the pharmacokinetics issue. We suggest a treatment paradigm with two leukotriene modifiers zileuton CR/montelukast in individuals presenting with minor symptoms and receiving diagnosis of a positive test for SARS-CoV-2. The drugs would be administered orally for a period of approximately 1–3 weeks until symptoms resolve completely and diagnosis indicating negative for SARS-CoV-2 infection. This regimen could be added, in theory, as a trial arm to an existing protocol (similar to the Phase III COSMO trial) or as a standalone trial to avoid the potentially fatal hyperinflammatory response.

## Discussion

The rationale for the role of leukotrienes in COVID-19 pathogenesis is clear. A wealth of information exists on elevated LTs in ARDS/sepsis/end organ failure in humans and efficacy of both zileuton and montelukast in various preclinical models (Sprague et al., 1989; Davis et al., 1990; Bernard et al., 1991; Westcott et al., 1991; Collin et al., 2004; Khodir et al., 2014; Monteiro et al., 2014). The roles of LTs in COVID-19 have been neglected to date. To strengthen the case for LT involvement in COVID-19 severity, non-invasive measurements of the major urinary metabolite of cysLTs, known as LTE_4_ (Sala et al., 1990), should be performed in normal controls and people infected at various stages with SARS-CoV-2 (**Figure 1**), as previously executed in ARDS patients and controls (Bernard et al., 1991; Sala et al., 1991; Westcott et al., 1991). By targeting vascular permeability, immune modulating and general inflammation-dampening effects at the CysLT_1_ level with montelukast (Dahlén et al., 1981; Maeba et al., 2005; Capra et al., 2007; Tahan et al., 2008; Khodir et al., 2014) and LT biosynthesis with the 5-lipoxygenase inhibitor zileuton, to block both arms of the LT pathway (**Figure 2**) and remove ligands for another key receptor regulating vascular permeability, CysLT_2_ (Moos et al., 2008), as well as inflammatory cell recruitment and endothelial cell adhesion *via* BLT_1_ receptor (Ford-Hutchinson et al., 1980; Tager et al., 2003; Taube et al., 2006; Sasaki and Yokomizo, 2019), there is a sound scientific basis for alleviating disease progression from mild to severe-critical stages of COVID-19 (**Figures 1** and **2**).

Both drugs are generic. While montelukast is inexpensive (about $1/day), the zileuton CR formulation is rather expensive (about $100 day), only available in certain countries (e.g., USA, but not in Canada), and requires a sophisticated process to manufacture the CR/instant release formulation. This pricing is still much less than a 5-day course of the antiviral drug remdesivir (over $3,000 USD)^6^, which has shown limited efficacy to date (Goldman et al., 2020). Drug-drug interactions may have to be monitored (liver function tests). While zileuton is metabolized primarily *via* CYP1A2 and montelukast *via* CYP2C8, both are metabolized by the same secondary CYP450 liver enzymes (e.g., 2C9, 3A4) (Funk, 2005). Since the treatment paradigm we are proposing is a single, short-term treatment, hepatic function monitoring should not be a major concern since hepatotoxicity, when it does occur, usually happens after a month and is infrequent with zileuton alone^7^. The timing of drug administration during the clinical course of COVID-19 is important, especially so, for a general immune system dampening treatment like dexamethasone, which interestingly may also diminish leukotriene production by virtue of its effects to block substrate supply *via* the enzyme cytosolic phospholipase A_2_ (Yao et al., 1999). The initial host antiviral immune response to SARS-CoV-2 involves steps of innate immunity implicating interferons (Zhou Q. et al., 2020), so dexamethasone may quell the initial host antiviral response. Leukotrienes, while generally considered “villain” inflammatory mediators, are in some contexts of pathogen invasion deemed innate effectors of the immune response but not for all cases in pre-clinical models (Secor et al., 1998; Peters-Golden et al., 2005; Flamand et al., 2007). The timing for zileuton/montelukast administration in humans with COVID-19 should be addressed.

Montelukast has been predicted to bind to the SARS-CoV-2 main protease (MPro) and could perhaps disrupt viral replication (Almerie and Kerrigan, 2020; Wu C. et al., 2020). A coordinated network of lipid signaling molecules including LTs, as well as cytokines, orchestrates proper leukocyte recruitment in settings of inflammation (Sadik and Luster, 2012). In COVID-19, providing the zileuton/montelukast combination prior to out-of-control host inflammatory cell recruitment to the lungs and before pulmonary edema sets in is paramount. Whether females would benefit preferentially from LT modifiers compared to males due to known androgen-dependence of leukotriene biosynthesis (Pergola et al., 2008) and preclinical effects noted with a non-approved member in this class in other settings (Pace et al., 2017) remains to be determined. We are recommending initiation of treatment with zileuton/montelukast in the 24-48 h window when a positive test is confirmed and before major symptoms arise, if feasible. The impetus to move forward quickly is vital to combat SARS-CoV-2 while waiting for a preventative vaccine (Funk et al., 2020) or other treatments such as neutralizing antibodies.

## Data Availability Statement

All relevant data is contained within the article. The original contributions presented in the study are included in the article, further inquiries can be directed to the corresponding author.

## Author Contributions

CF and AA conceived the design and concepts. CF wrote the manuscript. Both authors contributed to the article and approved the submitted version.

## Funding

This research was funded by Novateur Ventures, Inc.

## Conflict of Interest

AA is the founder and managing director of Novateur Ventures, Inc. CF is a scientific advisor for Novateur Ventures Inc. The authors have applied for a provisional patent for this particular drug combination to treat COVID-19. The authors currently have no commercial or financial relationships with the makers of the two drugs discussed in this article.

## Footnotes

1 https://coronavirus.jhu.edu/map.html [accessed June 30, 2020]

2 https://www.aaaai.org/conditions-and-treatments/drug-guide/leukotriene-modifiers [accessed June 29, 2020]

3 https://clinicaltrials.gov/ct2/results?cond=COVID-19 [accessed June 29, 2020]

4 https://public.tableau.com/profile/marinamarin#!/vizhome/covidTrials/COVID-19ClinicalTrialsExplorer [accessed June 29, 2020]

5 https://clinicaltrials.gov/ct2/show/NCT04389411 [accessed June 29, 2020]

6 https://endpts.com/gilead-opts-for-3120-us-insurer-price-for-covid-19-drug-remdesivir-let-the-debate-begin/ [accessed June 30]

7 https://www.ncbi.nlm.nih.gov/books/NBK548397/ [accessed June 29, 2020]

## Abbreviations

COVID-19, Coronavirus disease 2019; SARS-CoV-2, Severe acute respiratory syndrome coronavirus 2; ARDS, acute respiratory distress syndrome; CRP, C-reactive protein; MIS-C, multisystem inflammatory syndrome in children; LT, leukotriene; cysLT, cysteinyl leukotriene; LTB_4_, leukotriene B_4_; BLT_1_, B leukotriene receptor subtype 1; CysLT_1_, cysteinyl leukotriene receptor subtype 1; CysLT_2_, cysteinyl leukotriene receptor subtype 2; CSS, cytokine storm syndrome; GPCR, G protein-coupled receptor; LTRA, leukotriene receptor antagonist; CR, controlled release.
